# Biopolymer colloids for controlling and templating inorganic synthesis

**DOI:** 10.3762/bjnano.5.222

**Published:** 2014-11-17

**Authors:** Laura C Preiss, Katharina Landfester, Rafael Muñoz-Espí

**Affiliations:** 1 Max Planck Institute for Polymer Research, Ackermannweg 10, 55128 Mainz, Germany

**Keywords:** biomacromolecules, biopolymer, colloid, nanoparticle, organic–inorganic hybrid, template

## Abstract

Biopolymers and biopolymer colloids can act as controlling agents and templates not only in many processes in nature, but also in a wide range of synthetic approaches. Inorganic materials can be either synthesized ex situ and later incorporated into a biopolymer structuring matrix or grown in situ in the presence of biopolymers. In this review, we focus mainly on the latter case and distinguish between the following possibilities: (i) biopolymers as controlling agents of nucleation and growth of inorganic materials; (ii) biopolymers as supports, either as molecular supports or as carrier particles acting as cores of core–shell structures; and (iii) so-called “soft templates”, which include on one hand stabilized droplets, micelles, and vesicles, and on the other hand continuous scaffolds generated by gelling biopolymers.

## Introduction

During the natural synthesis of inorganic matter in living organisms, referred to as biomineralization, biogenic macromolecules are not only present in the crystallization medium, but play a crucial role in the mineral formation. Biomacromolecules, (e.g., polysaccharides, proteins, and nucleic acids) can have thereby two main functions: (i) a controlling effect on nucleation and growth of the inorganic material, and (ii) a structuring function, either confining spaces or acting as supports or as scaffolds for the growth. As a result of the interaction of organic and inorganic matter, nature is able to create hybrid materials whose exquisite structures and properties continue to impress humankind [1]. Egg shells, nacre, corals, or biosilica in sponges are still nowadays fascinating materials for scientists, who try to imitate natural strategies in the laboratory with only limited success.

It is clear that all synthetic routes based on the use of (bio)polymers as controlling and templating agents in inorganic synthesis have in one or other way their origin or inspiration in natural strategies. We do not wish, however, to insist once more on the well-known ditty on the use of nature as “bioinspiration” for science. Our aim, probably more modest, is to classify and review here some of the recent – and in our opinion most representative – synthetic works involving the use of biopolymer and biopolymer colloids for the design of inorganic and inorganic/organic materials, with special emphasis on particles and particle synthesis.

In the formation of polymer/inorganic hybrid materials, both the inorganic and the polymer component can be formed either in situ or ex situ (i.e., prepared independently before the formation of the hybrid final material), leading to four combinatorial possibilities: in situ/in situ*,* in situ/ex situ*,* ex situ/ex situ, and in situ/in situ. These different strategies for the formation of hybrid materials have been recently reviewed elsewhere in detail [2]. In the present review, we will describe the use of biopolymers as controlling agents and templates, which implies that the polymer is almost always formed beforehand. Nevertheless, cross-linking processes of the polymer can occur simultaneously to the inorganic precipitation/crystallization. With these considerations in mind, and centering our attention on the formation of the inorganic materials and not of the biopolymer, we should distinguish two possibilities:

1. Approaches in which the inorganic component is formed ex situ and later combined with the polymer
2. Approaches in which the formation of the inorganic material takes place in situ, that is, while the biopolymers are already present in the system

**Figure 1:**
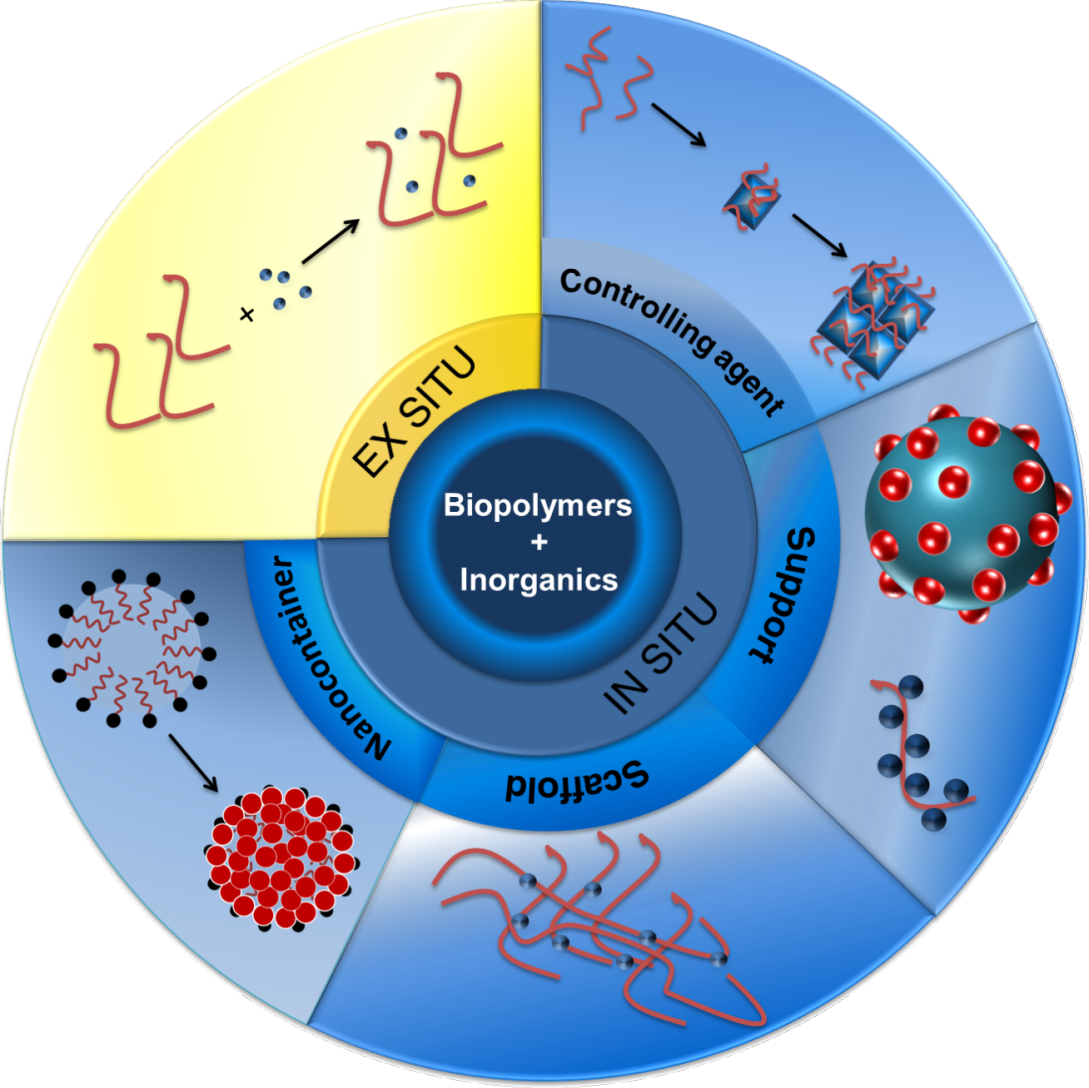
Different roles of biopolymers as controlling agents and templates in the formation of inorganic materials.

In the first situation, with the inorganic material being formed ex situ, biopolymers can probably be considered neither as controlling agents nor as templates in a strict sense, at least not for the synthesis. However, before entering to describe the in situ formation, we will briefly refer to the ex situ case for the sake of completeness. Figure 1 represents schematically the ways in which biopolymers can be useful for designing inorganic and inorganic/organic materials, including the ex situ synthesis and the different cases of the in situ formation, further classified in the corresponding section below.

## Review

### Ex situ formation of the inorganic material

Hydrogels, such as those based on the polyaminosaccharide chitosan, are probably the most commonly used scaffolds for the preparation of bipolymer/inorganic composites, very especially for biomedical applications. Aimé and Coradin have reviewed the topic in a recent publication [3]. By mixing colloidal inorganic particles formed ex situ with a biopolymer sol, followed by a gelation process, porous hybrid structures can be obtained. Starting from a colloidal suspension of laponite particles, Shi et al. [4] reported the preparation of a nanocomposite matrix of chitosan and clay that was applied as a glucose biosensor. Very recently, da Costa Neto et al. [5] prepared chitosan/silica composite microspheres by mixing an aqueous solution of the biopolymer with commercial nanosized silica particles. The obtained microparticles were dried afterwards. In further examples, chitosan matrices have also been used to immobilize CdSe quantum dots [6] and γ-Fe_2_O_3_ nanoparticles [7].

In a different approach, biopolymers can also be applied to modify surfaces and induce the deposition of nanoparticles. For instance, Nochomovitz et al. [8] described the deposition and patterning of gold colloidal nanoparticles and carbon nanotubes on surfaces previously modified with peptides.

### In situ formation of the inorganic material

After having briefly discussed a few examples in which the inorganic material is formed ex situ and combined a posteriori with biopolymers, we will revise now in situ strategies, with biopolymers playing an active role during the formation of inorganic materials. We propose the following classification, being aware that all divisions are arbitrary to some degree and it may be difficult to place some of the examples in one or other group without ambiguity:

A. The use of biopolymers and biopolymer colloids as controlling agents for the precipitation and crystallization of inorganic materials, which is typically referred to as “polymer-assisted” or “polymer-controlled” formation and is intimately related to the (bio)mineralization field.

B. Biopolymers as “supports” for precipitation/crystallization processes. We distinguish depending on whether the formation of inorganic nanoparticles takes places on biopolymer molecules or on particles: (B1) Nanoparticle formation on biopolymer molecules (often referred to as “metallization” and “mineralization” of biopolymers). (B2) Biopolymer particles as support, with the formation of the inorganic nanoparticles taking place on the surface.

C. Biopolymers as so-called “soft templates”. Differently from the previous case, here the precipitation/crystallization of the inorganic materials does not take place merely on the surface, but *within* the supramolecular structure formed by the biopolymer (polymer matrix). Among the “soft templates”, two subgroups can be considered: (C1) Biopolymer-stabilized spherical geometries (stabilized droplets, micelles, and vesicles) that confine the inorganic formation. (C2) Biopolymer structures acting as “scaffolds”, with more complex geometries than simple spheres. This is typically the case for gels and microgels. Microgels can also be prepared in the form of nanoparticles, which can be considered a kind of intermediate case between C1 and C2.

### A. Biopolymers and biopolymer colloids as controlling agents: polymer-controlled crystallization

Many types of polymers, both of natural origin and synthetic, have been used as controlling agents for crystallization. This field of the so-called “polymer-controlled crystallization” has been reviewed in detail in several publications of Cölfen and collaborators [9–12].

Among the different natural or biomimetic polymers studied, we find starch [13–14], different cellulose derivatives [15], dextran [16], pectin [17], alginate [18], and poly(amino acids) or proteins [19–29]. Researchers in the biomineralization field very often extract proteins from biological matter and use them for the ex vivo mineralization, trying to study the effects of natural macromolecules [30]. Silicateins, for instance, are proteins not only used ex vivo for understanding mineralization processes in sponges, but also applied to prepare novel biomimetic hybrid materials, as nicely revised in a recent publication by Müller et al. [31].

From the mineral side, the most investigated systems are by far the calcium minerals because of their biological importance: calcium carbonate [16–18,20,32], calcium oxalate [23–26,33], and calcium phosphates (including hydroxyapatite) [22]. Nevertheless, biopolymers have also been used as controlling agents or additives in the precipitation/crystallization of other inorganic systems, such as ZnO [34], metal particles [13], silica [35], or Fe_2_O_3_ [15].

**Figure 2:**
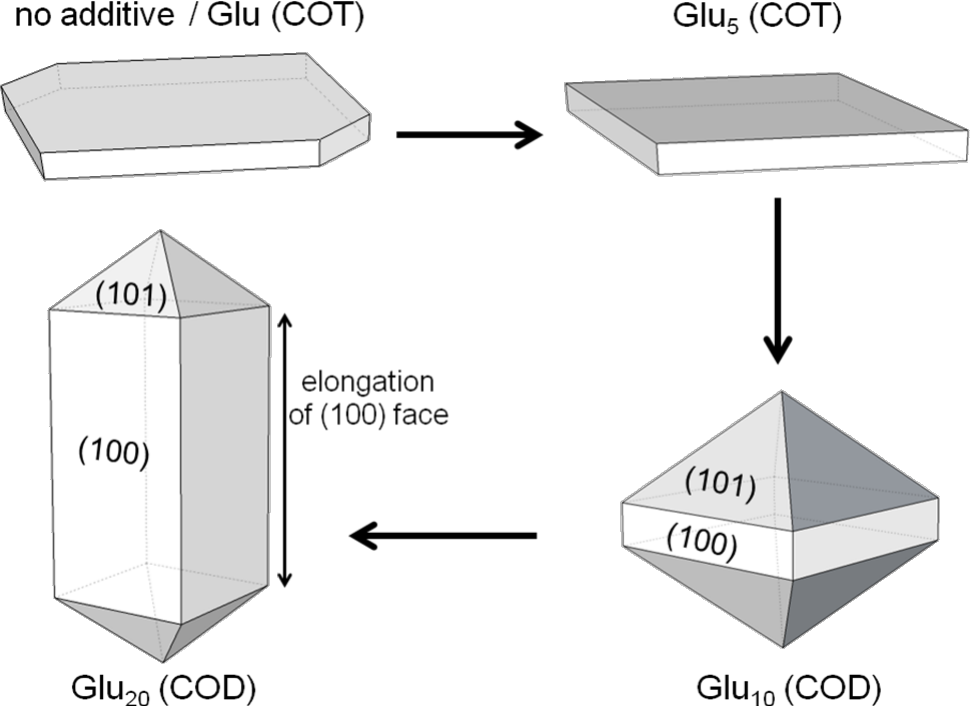
Schematic representation of the evolution of the morphology of calcium oxalate crystals prepared in the presence of oligo(L-glutamic acid)s of different lengths, ranging from the monomer (Glu) to the eicosamer (Glu_20_). COD: calcium oxalate dihydrate; COT: calcium oxalate trihydrate. Reprinted with permission from [38]. Copyright 2010 American Chemical Society.

To investigate the effects of proteins in mineralization, synthetic oligopeptides with sequences of defined lengths and composition are sometimes used [23–24,36–37]. A previous work from our research group showed that an increasing length of oligo(L-glutamic acid) chains is able to change not only the morphology of the obtained crystals, but also to stabilize the metastable calcium oxalate dihydrate (Figure 2) [38]. In a more recent work, we have also shown that charged acidic peptides are able to stabilize vaterite, and we studied the effect of the acidity of the amino acid residues on this stabilization [39].

In a previous work, synthetic polymer colloidal particles functionalized with different groups were shown to have an effect on the growth and on the final properties of inorganic materials such as zinc oxide [40–41], calcium oxalate [38], or calcium carbonate [42–43]. It is expectable that analogous effects should be obtained when biopolymeric (or synthetic biomimetic chains) are attached to the surface of colloidal particles. In this sense, Krattiger et al. [44] reported the morphogenesis of CaCO_3_ and DL-alanine crystals in the presence of polystyrene beads functionalized with synthetic peptides with different amino acids and oligopeptides.

### B. Biopolymers as “supports”

**B1. Molecular templates:** Biomacromolecules contain often functional groups (such as phosphates in DNA or carboxylic and amino groups in proteins) that are able to complex metal ions and act as nucleation centers for the growth of metal or mineral nanoparticles. The use of molecular templates as a support for inorganic nanoparticles may be referred to as “metallization” or “mineralization” (depending on whether metal or mineral particles are formed) of biopolymers. Zinchenko [45] reviewed the advances in the field, with special emphasis on DNA and its assemblies, but going also through the use of proteins. Although there are common points between such molecular templating and the polymer-controlled crystallization described above, and in some cases the distinction may be unclear, the main difference lies on the size of the formed particles and the polymer. In the case of molecular supports, tiny inorganic nanoparticles are formed on the biomacromolecular chain, while in polymer-controlled crystallization processes the inorganic material is significantly larger than the macromolecules, which may get engulfed by the growing crystals.

**Figure 3:**
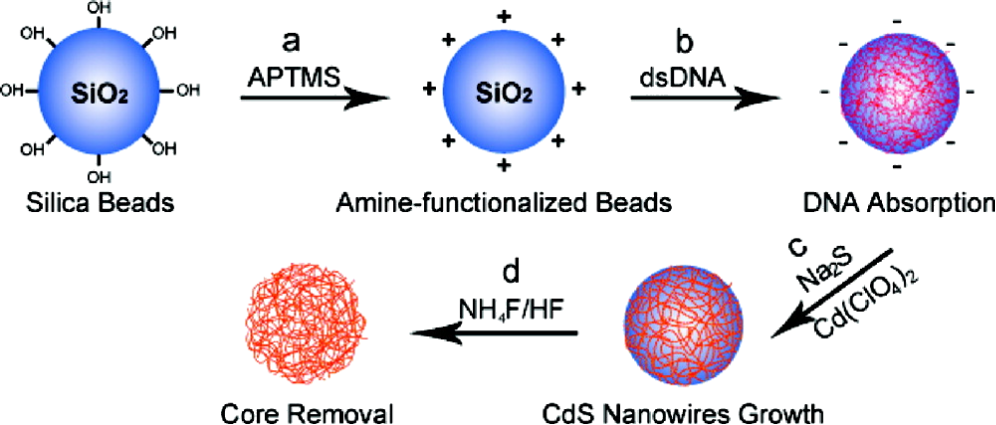
DNA-templated preparation of porous CdS shells on the surface of silica beads: (a) surface modification with (3-aminopropyl)trimethoxysilane (APTMS), (b) DNA deposition on the cationic particle surface, (c) CdS precipitation, and (d) dissolution of the SiO_2_ core to form hollow structures. Reprinted with permission from [52]. Copyright 2011 American Chemical Society.

DNA chains have been coated by in situ deposition with different metals, metal oxides, and metal chalcogenides, including metallic silver [46], Pt [47], Fe_2_O_3_ [48], and CdS [49–52]. Pu et al. [52] reported the deposition of DNA chains on silica particles. After mineralization of the DNA to CdS as shell and subsequent removal of the silica core by dissolution with HF, hollow inorganic particles were obtained (Figure 3). Analogous to the DNA case, peptidic supports have also been used for the deposition of metals [53] and semiconductor chalcogenide quantum dots [54–65].

**B2. Biopolymer particles as “supports”:** In the area of preparation of hollow particles it is common to distinguish between “hard” and “soft” templates [57–59]. This nomenclature can also be extended to the formation of polymer/inorganic particles. “Soft templates” will be reviewed in Section C; here, we will consider the case of so-called “hard templates”, which typically involves the deposition of an inorganic material on the surface of “hard” spheres (silica or polymer) that act as sacrificial cores. The core can be eventually removed by calcination or dissolution, if the aim is the formation of hollow structures. Such strategies have been widely used for templates with synthetic polymers (see Section 4 in [60] for a review), but only a limited number of works are found for biopolymers.

**Figure 4:**
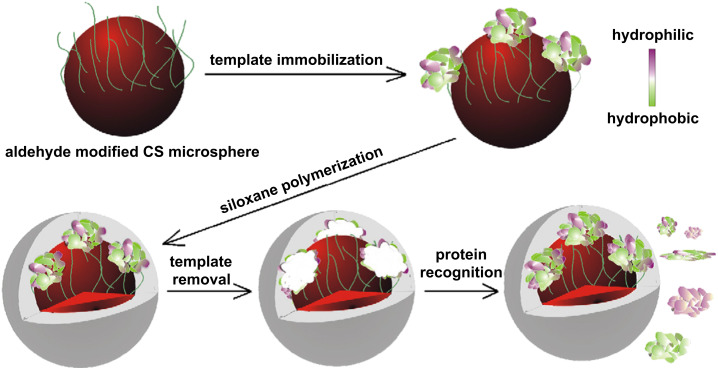
Siloxan polymerization on chitosan microspheres by using immobilized protein templates. Reprinted with permission from [61]. Copyright 2008 Elsevier.

Li et al. [61] prepared cross-linked chitosan microspheres and immobilized bovine serum albumin covalently on their surface. On the resulting particles, silica was formed by a sol–gel process from 3-aminopropyltrimethyoxysilane (APTMS) or tetraethoxysilane (TEOS). The structures after the removal of the template were proven to be suited for protein recognition. The synthesis process is depicted in Figure 4.

When referring to polymer particles, the denomination “hard template” may sound somehow odd, even more in the case of microgel particles, which are definitely not “hard”. However, in our classification we consider within this group all approaches in which a shell is formed on the surface of a particle. As an example, Boissière et al. [62] synthesized poly(L-lysine)/alginate microparticles through a microgel route and coated them with silica to obtain core–shell composites. In an alternative method, spray-drying of biopolymer and biopolymer/silica solutions was conducted. Magnetic cobalt silicate could be also generated by introducing a cobalt salt during the process.

### C. Biopolymers as “soft templates”

**C1. Biopolymer-stabilized simple geometries (droplets, micelles, and vesicles):** Surface-active polymers can assemble in solution and in heterophase systems to form defined geometries, most typically spherical, such as micelles, vesicles, or even stabilized droplets. As in the case of “hard templates” stated above, the approach has been very productive with synthetic polymers [2,60], but only explored in a limited way with biopolymers. The main reason for this is that the assembly of many biopolymers results in a rather continuous network and not necessarily in “discrete” geometries.

**Figure 5:**
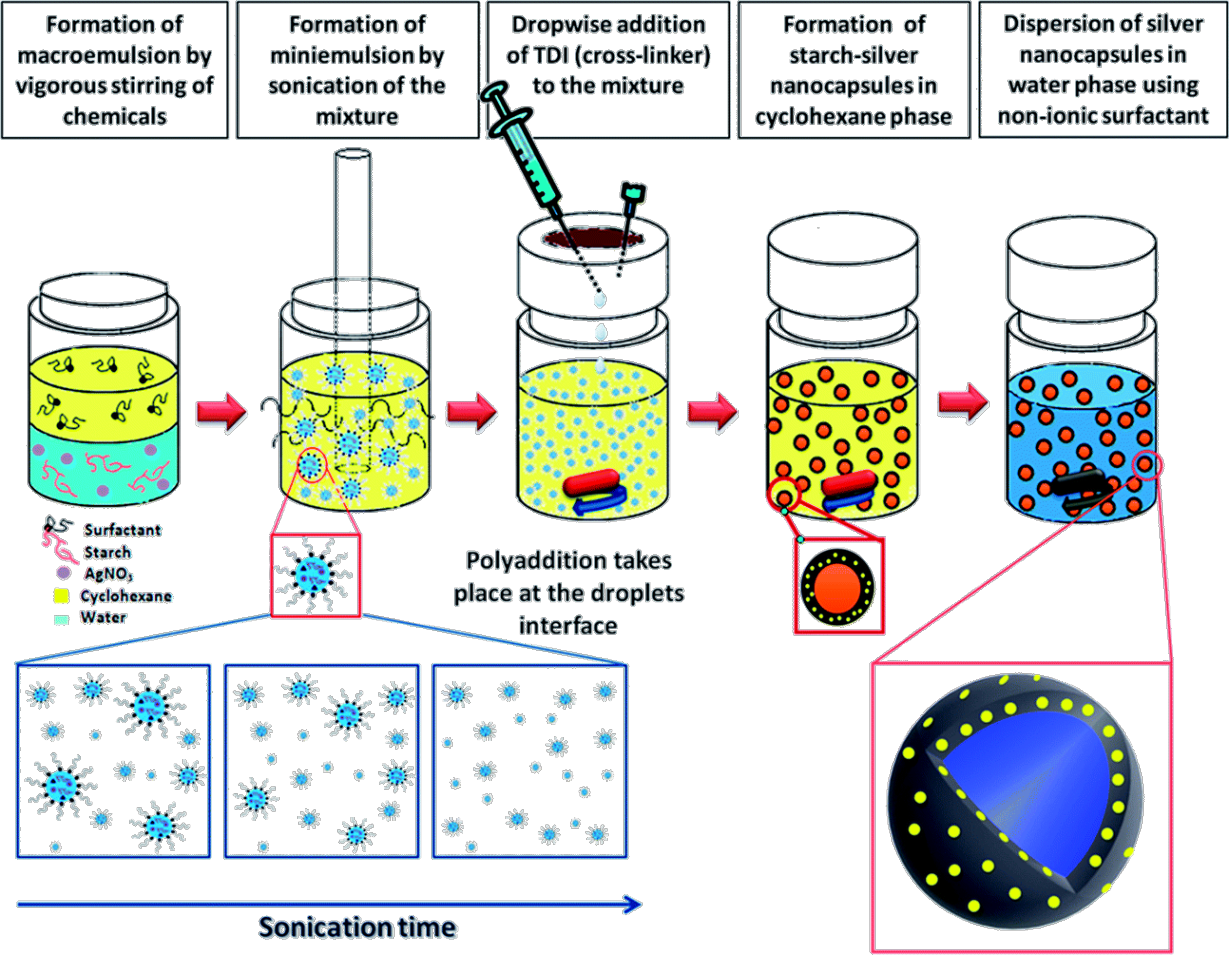
Schematic representation of the procedure applied for synthesizing starch/Ag nanocapsules. Reprinted with permission from [63]. Copyright 2014 The Royal Society of Chemistry.

In a very recent work, Taheri et al. [63] have presented the formation of potato starch capsules decorated with silver nanoparticles, which could have applications as drug carriers or antibacterial coatings. The capsules are prepared in an inverse (water-in-oil) miniemulsion and the surfactant polyglycerine-polyricinoleate (PGPR) is used to stabilize the system. Interestingly, a polyaddition process of the starch, driven by the addition of 2,4-toluene diisocyanate (TDI), occurs simultaneously to the reduction of Ag^+^ ions to metallic silver without addition of any additional reducing agent (Figure 5). Since polyaddition and silver precipitation occur both at the same time, the approach could be considered as an “in situ/in situ” or “all in situ” strategy, which is a rather rare case in literature. We have decided to include it within this subsection mainly due to the spherical geometry and the presence of a heterophase system, but probably the example could have been included as well in the next subsection, as the silver precipitation takes place within the polymer scaffold.

**Figure 6:**
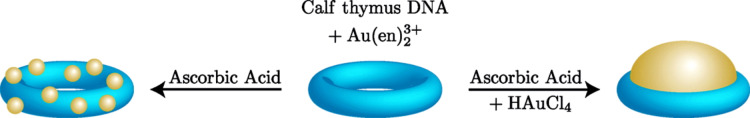
Products obtained when gold(III) is reduced in the presence of DNA toroids formed with bis(ethylenediamine)gold(III). Reprinted with permission from [66]. Copyright 2010 American Chemical Society.

As an extension of the metallization examples presented in Section B1, structures formed by DNA or proteins can also be used for templating. The toroidal structures formed by DNA condensates were used as soft templates for the formation of silver [64–65] and gold [66] nanoparticles by reduction of the metal salts (Figure 6). Rings resulting from the assembly of a bolaamphiphilic peptide molecule were reported as templates for the growth of conductive indium tin oxide (ITO) nanoparticles [67].

**C2. Biopolymers as “scaffolds”:** As mentioned above, gelling biopolymers are very common templates in inorganic syntheses [3,68]. Since the precipitation/crystallization of the inorganic nanoparticles takes place within the network generated by the polymer and not on the surface or edges (as it is the case of the “supports” of the previous subsection), we label this type of templating as “scaffold”, being aware that the term is also used in a more general way – almost as a simple synonym for “template” – by other authors.

Because of the biodegradability and biocompatibility, chitosan can be considered as a “green material”. In addition to the common applications in food and biotechnology, chitosan can also be used as a support for catalysts. Chitosan–silica [69] and chitosan–titania [70] catalysts were prepared by applying conventional sol–gel methods. The preparation of sol–gel silicates have been reported by several research groups [71–72]. Nevertheless, the use of chitosan is not limited to silicates and titanates. El Kadib et al. [73] demonstrated the use of chitosan microspheres as templates for vanadium, tungsten, and molybdenum oxide clusters, which were shown to be active as catalysts for selective alcohol oxidation. Similarly, Ganesan and Gedanken [74] had prepared tungsten(VI) oxide nanoparticles through the encapsulation of ammonium metatungstate on chitosan and the subsequent calcination. These particles showed a higher catalytic activity than bulk tungsten trioxide. Other materials, such as cobalt-Prussian blue nanoparticles [75], Zn–Al layered double hydroxide [76], hydroxyapatite [77], and calcium carbonate [78], were also prepared within, or in the presence of, chitosan gels. In a biological approach, calcium phosphate/chitosan composite films were shown to influence the behavior of human mesenchymal stem cells. Lee et al. [79] studied the scaffold–cell interaction by changing the crystallinity and ratio of the calcium phosphate.

Alginate is another of the gelling biopolymers used as a scaffold. An alginate-influenced growth of Co, Ni, and CoNi nanoparticles was reported by Coradin et al. [80]. The same research group also studied the in situ growth of gold colloids with alginate films [81–82]. Gel frameworks have been shown to be able to control the size distribution of particles. Hernández et al. [83] demonstrated the synthesis of iron oxide nanoparticles in a semi-interpenetrating polymer network of alginate and poly(*N*-isopropylacrylamide).

**Figure 7:**
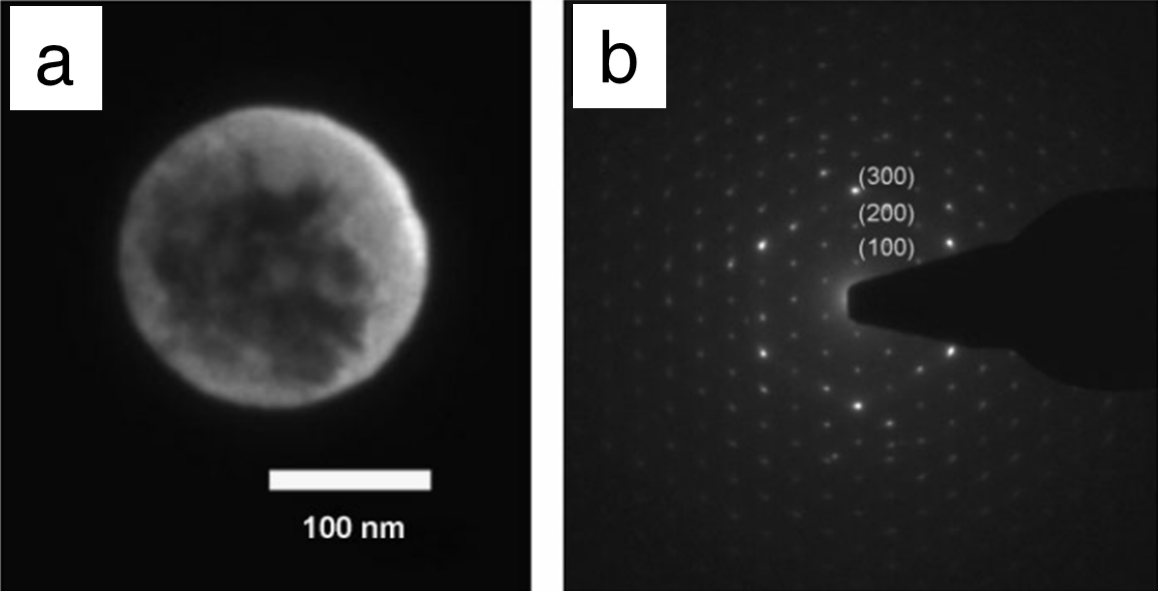
Dark-field TEM micrograph (a) and corresponding electron diffraction pattern (b) of hydroxyapatite/gelatin particles. Reprinted with permission from [87]. Copyright 2008 John Wiley & Sons.

Gold and AuNi alloy gelatin nanocomposites were developed by Brayner et al. [84]. A gelatin network incorporating metallic nanoparticles was obtained after reduction of gold salts. Like other gel biopolymer templates, gelatin has also been used in silicate sol–gel processes [72,85–86]. Ethirajan et al. [87] used the confinement provided by gelatin particles prepared through a miniemulsion to template the crystallization of hydroxyapatite (Figure 7).

**Figure 8:**
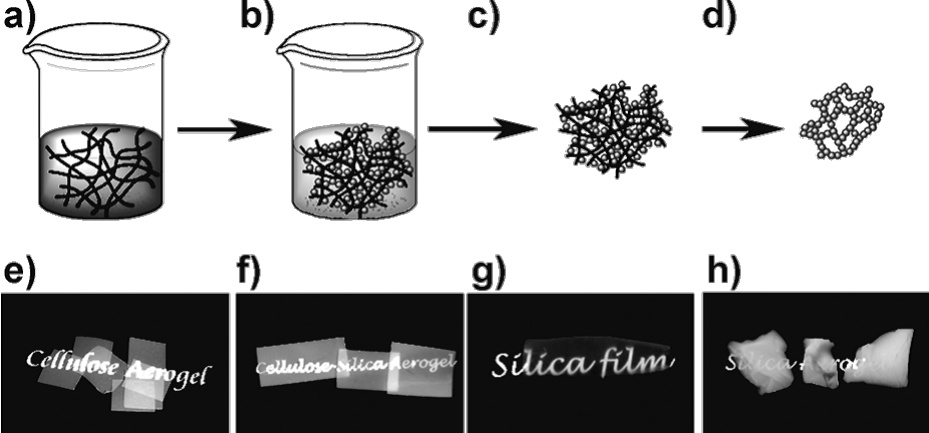
Schematic representation of aerogel preparation. A nanoporous cellulose gel is impregnated with the silica precursor TEOS (a). Afterwards, the silica formation takes place by sol–gel process (hydrolysis and condensation), yielding a cellulose–silica composite gel (b). Drying with supercritical CO_2_ gives a composite aerogel (c). Macroscopic views of the prepared samples are shown in panels (e–h). Reprinted with permission from [90]. Copyright 2012 John Wiley & Sons.

A further example of a heterogeneous catalyst was reported by Taubert’s group with gold/cellulose nanocrystal hybrids produced in the presence of ionic liquids [88]. Also for catalytic applications, nanoparticles of silver, gold, and platinum were synthesized by using a cellulose aerogel [89]. Cellulose has been further used for silicates. Zhang et al. [90] presented the in situ formation of silica in a cellulose aerogel (Figure 8). The addition of the silicate precursor (TEOS) takes place first, followed by a sol–gel process and the cellulose/silica composite formation. The aerogel is formed by drying with supercritical CO_2_ and subsequent calcination.

Scaffold templating can also be achieved with starch and even with peptides. Thakore et al. [13] synthesized Cu, Ag, and Cu–Ag alloy nanoparticles in a matrix of starch through a green route and studied the antibacterial activity. Hexagonal silica platelets were prepared through a polypeptide-templated synthesis by using the interactions of a polypeptide of L-lysine with silicate [35].

## Conclusion

The application of biopolymers (polysaccharides, peptides, and nucleic acids) as controlling agents or as templates of inorganic precipitation and crystallization is not only present in nature (biomineralization), but is also a versatile strategy for the design of inorganic and inorganic/organic hybrid materials in the laboratory. On one hand, biopolymers may assemble forming structures that serve as confining spaces or scaffolds in which the formation of the inorganic component takes place. On the other hand, the presence of functional groups such as carboxylic, amino or phosphate groups can provide a high ability to bind metal ions or to interact with growing crystal faces, influencing nucleation and growth.

Hydrogels, such as those formed with chitosan or gelatin, are very commonly used as polymer matrices for the synthesis of porous structures. Although in general the inorganic material is formed in situ (i.e., while the biopolymer is present in the system), there are also some examples in which previously formed nanoparticles are combined with the biopolymer and incorporated into the matrix after gelation.

In most cases, biopolymers have been either used in bulk solutions or applied to surfaces, so that the resulting material is a continuous hybrid structure. However, they can also be used to generate “discrete” structures either by using single molecular chains as supports (e.g., metallization or mineralization of DNA) or by using particle systems. Hydrogel approaches can also be confined to the spaces of particles.

Clearly, synthetic polymers are often a more economic and versatile alternative, but biopolymers can be especially interesting in those applications in which biocompatibility or biodegradability are an issue, such as biomedical applications. In addition, biopolymers may be also good model systems. In this sense, for instance, peptides or nucleic acids of defined length and structure can be very convenient models for studying polyelectrolyte systems. Furthermore, the high ability of biopolymers to form complex hierarchical structures is a major feature to be explored in the upcoming years. A better understanding of the interface between the biopolymeric component and the growing inorganic matter will continue to be the crucial issue in the design of novel and more sophisticated materials.
